# Supplementary material for: Skilful decadal-scale prediction of fish habitat and distribution shifts
Source: Nat Commun. 2022 May 12;13:2660. doi: 10.1038/s41467-022-30280-0 (PMC9098506; doi:10.1038/s41467-022-30280-0)
Supplement: Supplementary file 1 — Supplementary Information [file 41467_2022_30280_MOESM1_ESM.pdf]

Supplementary Material for "Skilful decadal-scale prediction of fish habitat and distribution shifts "

Mark R. Payne[1,2] *, Gokhan Danabasoglu[3], Noel Keenlyside[4,5] Daniela Matei[6], Anna K. Miesner[7], Shuting Yang[1] and Stephen G. Yeager[3]

* Corresponding author

1. Danish Meteorological Institute, Copenhagen, Denmark
2. National Institute of Aquatic Resources (DTU-Aqua), Technical University of Denmark, Kgs. Lyngby, Denmark.
3. National Center for Atmospheric Research, Boulder, CO, USA.
4. Geophysical Institute, University of Bergen and Bjerknes Centre for Climate Research, Bergen, Norway.
5. Nansen Environmental and Remote Sensing Center, Bergen, Norway.
6. Max Planck Institute for Meteorology, Hamburg, Germany.
7. Helmholtz-Zentrum Hereon, Institute of Coastal Systems - Analysis and Modeling, Geesthacht, Germany

Corresponding author.
Mark R. Payne
Danish Meterological Institute
2100 Copenhagen
Denmark
ORCID: 0000-0001-5795-2481
E-mail address: mapa@dmi.dk

This PDF includes

- Supplementary Figures 1-3
- Supplementary Tables 1-3
- References

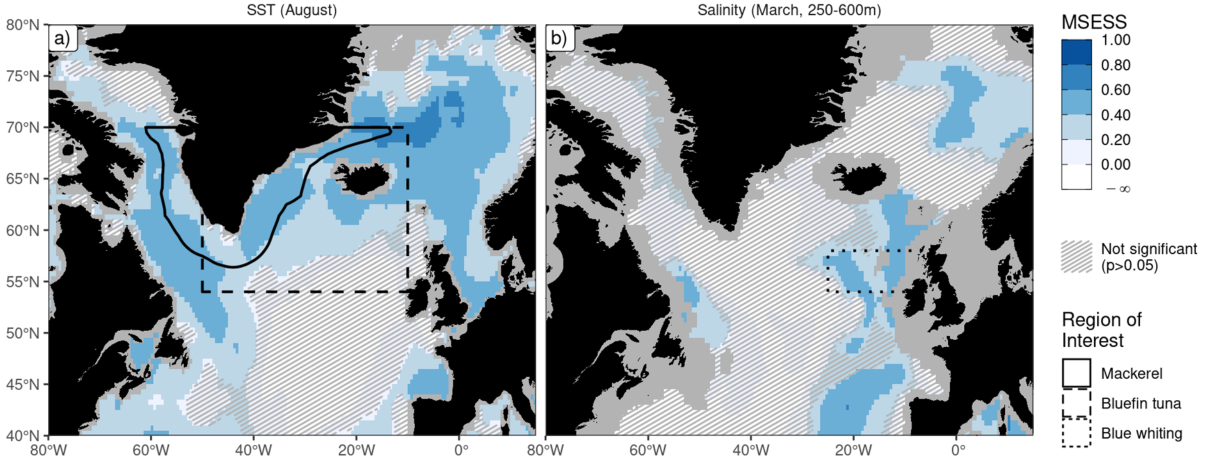

Supplementary Figure 1| Forecasts of distribution drivers skilfully predict the absolute value. As for Fig.1 but showing mean squared-error skill score (MSESS) (rather than Pearson correlation) as a measure of forecast skill. Predictive skill of physical variables underlying habitat forecasts showing a) sea surface temperature (SST) in August and b) sub-surface (250-600 m) salinity in March with a lead time of five years. Each grid point is coloured according to the local MSESS estimate. Forecast skill is for the grand ensemble mean forecast, i.e., averaged across the individual realisations from all model systems, covering the period 1960-2018 for SST and 1985-2018 for salinity. Regions where the MSESS is not significantly greater than 0 (at the 95% confidence level, as estimated from bootstrapping) are cross-hatched. Lines mark the polygons over which the area of suitable habitat is calculated in subsequent analyses.

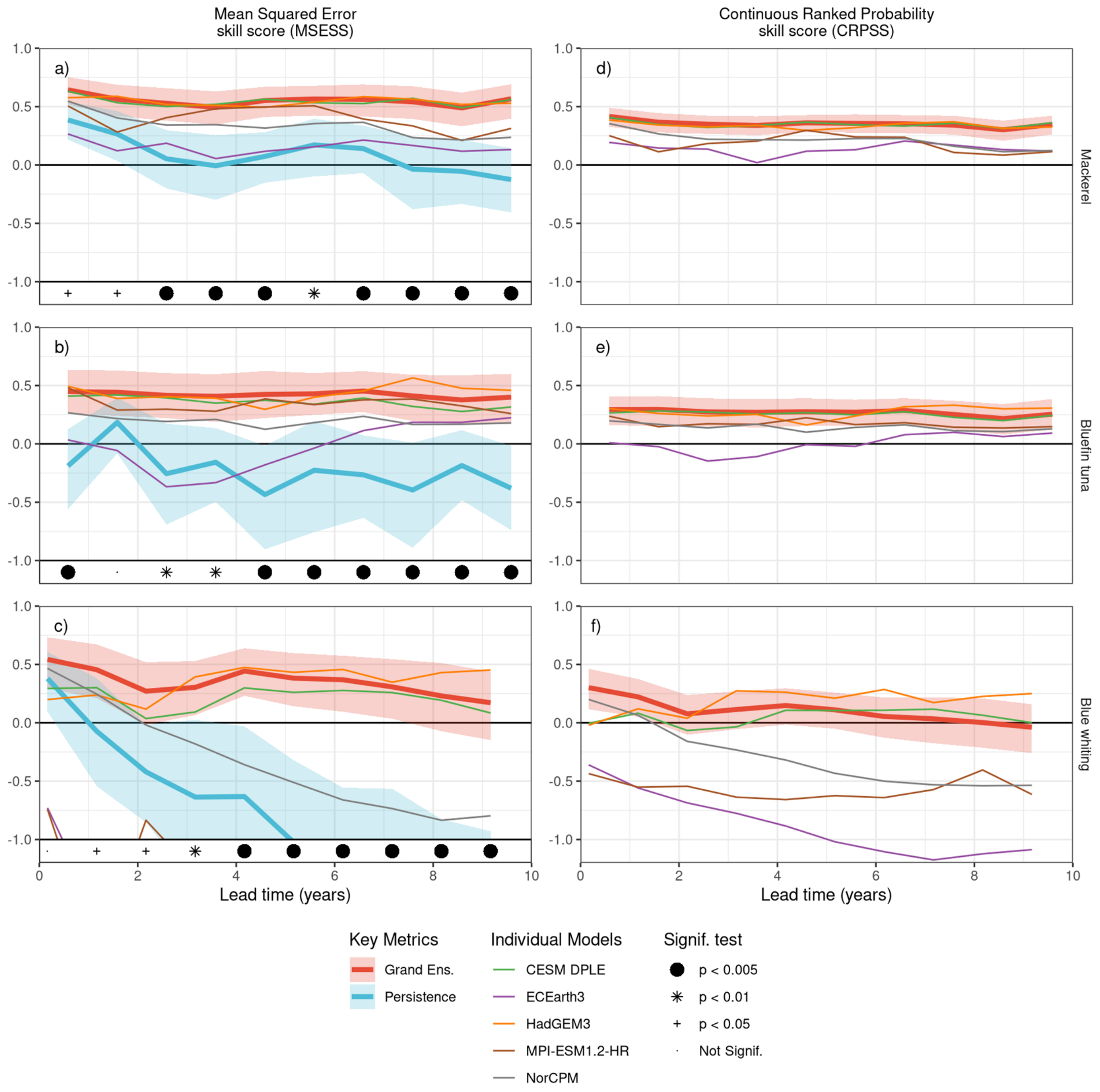

Supplementary Figure 2| The absolute values and probabilistic distributions of habitat metrics can also be skilfully forecast. As for Fig. 2 but showing additional metrics of forecast performance. The ability to correctly estimate the absolute habitat area is indicated by the Mean Squared Error skill score (MSESS) (panels a-c), while the Continuous Ranked Probability skill score (CRPSS) (panels d-f) indicates the probabilistic skill of the forecast distribution. Skill is shown for the habitat area of mackerel (panels a and d), bluefin tuna (b and e) and blue whiting (c and f). Skill metrics between the forecast and observed habitat areas are plotted as a function of forecast lead-time into the future, calculated across the appropriate comparison periods. Forecast skill is shown for the individual members of the model ensemble (light weighted lines) and for the grand-ensemble forecast (heavy red line). The skill of persistence forecasts (heavy blue lines) are also shown for reference where it can be defined (i.e. for MSESS): shaded areas for both these key metrics denote the 90% confidence interval estimated from bootstrapping. The hypothesis that the ensemble mean forecast outperforms persistence (i.e. a one-tailed test) is tested for each lead time, and denoted with symbols at the bottom of the MSESS panels. It is not possible to define a CRPSS metric for a persistence forecast and therefore no such results (or significance tests) are presented here. Both MSESS and CRPSS skill scores are calculated relative to the climatological statistics of each metric.

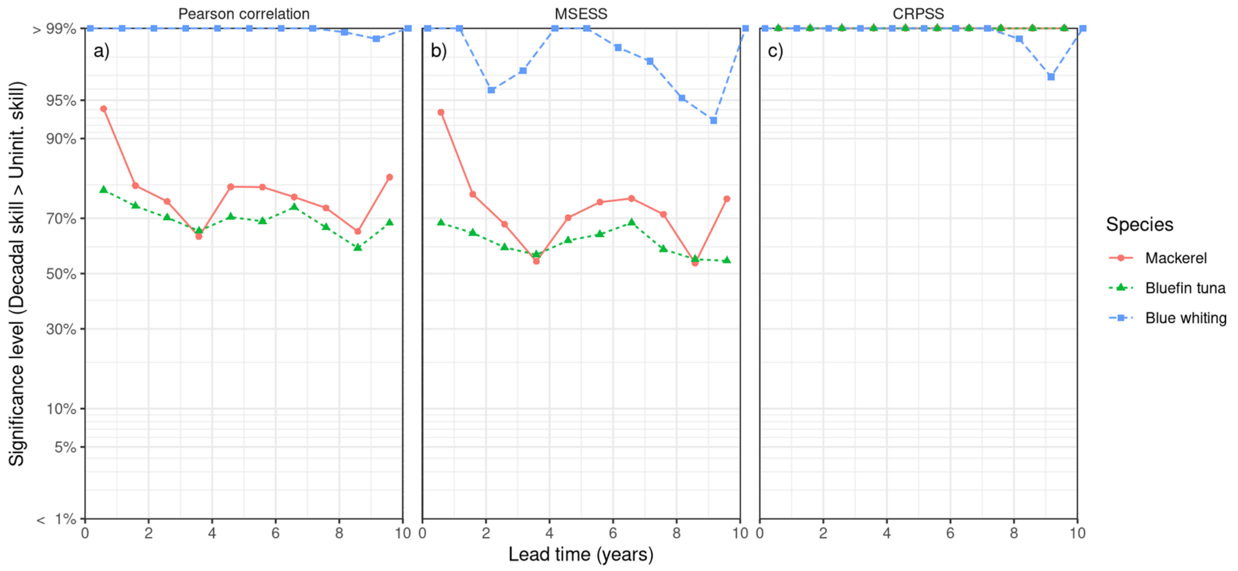

Supplementary Figure 3 | Habitat predictions from initialised climate models outperform forecasts based on uninitialized projections. The significance of habitat forecast skill when compared against the skill of habitat forecasts based on uninitialized forecasts (rather than persistence forecasts) for lead times of 0-10 years is shown for all species and for a) Pearson correlation coefficient, b) the mean-squared error skill score (MSESS) and c) continuous ranked probability skill scores (CRPSS). Significance levels (1 - p values) are plotted on the vertical axis for a one-sided test that the given skill of the decadal forecast system is greater than the uninitialized skill. Note the non-linear (probability) scale on the vertical axis. Significance levels outside the axis ranges are plotted at the top or bottom of each panel.

Supplementary Table 1| Habitat models used in this study

Species	Region	Environmental Variable and Month of Interest	Habitat model	References
Mackerel (*Scomber scombrus*)	Greenlandic exclusive economic zone, south of 70°N	Sea surface temperature in warmest month (August)	Suitable habitat is warmer than 11°C	[1,2]
Bluefin tuna (*Thunnus thynnus*)	Irminger Sea, Denmark Strait and waters south of Iceland.	Sea surface temperature in warmest month (August)	Suitable habitat is warmer than 8.5°C	[3–5]
Blue whiting (*Micromesistius poutassou*)	Rockall Trough and Rockall Bank, west of Great Britain and Ireland	Salinity between 250 and 600 m depth (March)	Statistical habitat model. Optimal salinity between 35.3 and 35.5 psu	[6–8]

Supplementary Table 2|Forecast systems and ensemble sizes used in this study

Forecast Centre	Model Name	Ocean Resolution	Start dates<br>Ensemble size	References
Bjerknes Center for Climate Research, Norway	NorCPM1	Tripolar, 1° grid, 53 vertical levels on density coordinates	1960-2018<br>20 members	[9]
Danish Meteorological Institute, Denmark	EC-Earth3	Tripolar 1° grid with meridional refinement down to 1/3° in the tropics; 75 levels	1960-2018<br>10 members	[10,11]
Max Planck Institute for Meteorology, Germany	MPI-ESM-1.2-HR	Tripolar, ~ 0.4° grid. 40 vertical levels.	1960-2018<br>5 members	[12,13]
Met Office Hadley Centre, UK.	HadGEM3-GC31-MM	Tripolar ~0.25° grid, 75 vertical levels	1960-2018<br>10 members	[14]
National Center for Atmospheric Research, USA	CESM DPLE	Nominal 1° horiz. with meridional refinement down to ~0.3° at the Equator; 60 vertical levels	1955-2018<br>40 members	[15,16]

Supplementary Table 3|CMIP6 models and representative variants used as uninitialized models. Ticks indicate that the given model, variant and gridded product were used for uninitialized forecasts of either sea surface temperature (SST) or salinity. In total, 35 models were used for salinity and 44 for SST.

Source ID	Institution ID	Variant Label	Grid Label	SST	Salinity
ACCESS-CM2	CSIRO-ARCCSS	r1i1p1f1	gn	✓	
ACCESS-ESM1-5	CSIRO	r1i1p1f1	gn	✓	
BCC-CSM2-MR	BCC	r1i1p1f1	gn	✓	✓
CAMS-CSM1-0	CAMS	r1i1p1f1	gn	✓	✓
CanESM5	CCCma	r1i1p1f1	gn	✓	✓
CanESM5-CanOE	CCCma	r1i1p2f1	gn	✓	✓
CAS-ESM2-0	CAS	r1i1p1f1	gn	✓	
CESM2	NCAR	r4i1p1f1	gn	✓	
CESM2-WACCM	NCAR	r1i1p1f1	gn	✓	
CESM2-WACCM	NCAR	r1i1p1f1	gr		✓
CIESM	THU	r1i1p1f1	gn	✓	✓
CMCC-CM2-SR5	CMCC	r1i1p1f1	gn	✓	✓
CMCC-ESM2	CMCC	r1i1p1f1	gn	✓	✓
CNRM-CM6-1	CNRM-CERFACS	r1i1p1f2	gn	✓	✓
CNRM-CM6-1-HR	CNRM-CERFACS	r1i1p1f2	gn	✓	✓
CNRM-ESM2-1	CNRM-CERFACS	r1i1p1f2	gn	✓	✓
EC-Earth3	EC-Earth-Consortium	r1i1p1f1	gn	✓	✓
EC-Earth3-CC	EC-Earth-Consortium	r1i1p1f1	gn	✓	✓
EC-Earth3-Veg	EC-Earth-Consortium	r1i1p1f1	gn	✓	✓
EC-Earth3-Veg-LR	EC-Earth-Consortium	r1i1p1f1	gn	✓	✓
FGOALS-f3-L	CAS	r1i1p1f1	gn	✓	✓
FGOALS-g3	CAS	r1i1p1f1	gn	✓	✓
FIO-ESM-2-0	FIO-QLNM	r1i1p1f1	gn	✓	✓
GFDL-CM4	NOAA-GFDL	r1i1p1f1	gn	✓	✓
GFDL-ESM4	NOAA-GFDL	r1i1p1f1	gn	✓	✓
GISS-E2-1-G	NASA-GISS	r1i1p1f2	gn	✓	✓
HadGEM3-GC31-LL	MOHC, NERC	r1i1p1f3	gn	✓	✓
HadGEM3-GC31-MM	MOHC	r1i1p1f3	gn	✓	✓
IITM-ESM	CCCR-IITM	r1i1p1f1	gn	✓	
INM-CM4-8	INM	r1i1p1f1	gr1	✓	✓
INM-CM5-0	INM	r1i1p1f1	gr1	✓	✓
IPSL-CM6A-LR	IPSL	r1i1p1f1	gn	✓	✓
KACE-1-0-G	NIMS-KMA	r1i1p1f1	gr	✓	
KIOST-ESM	KIOST	r1i1p1f1	gr1	✓	
MCM-UA-1-0	UA	r1i1p1f2	gn	✓	✓
MIROC-ES2L	MIROC	r1i1p1f2	gn	✓	
MIROC6	MIROC	r1i1p1f1	gn	✓	
MPI-ESM1-2-HR	MPI-M	r1i1p1f1	gn	✓	✓
MPI-ESM1-2-LR	MPI-M	r1i1p1f1	gn	✓	✓
MRI-ESM2-0	MRI	r1i1p1f1	gn	✓	✓
NESM3	NUIST	r1i1p1f1	gn	✓	✓
NorESM2-LM	NCC	r1i1p1f1	gn	✓	
NorESM2-LM	NCC	r1i1p1f1	gr		✓
NorESM2-MM	NCC	r1i1p1f1	gn	✓	
NorESM2-MM	NCC	r1i1p1f1	gr		✓
TaiESM1	AS-RCEC	r1i1p1f1	gn	✓	✓
UKESM1-0-LL	MOHC, NERC, NIMS-KMA, NIWA	r1i1p1f2	gn	✓	✓

References

1. Jansen, T. *et al.* Ocean warming expands habitat of a rich natural resource and benefits a national economy. *Ecol. Appl.* **26**, 2021–2032 (2016).

2. Nikolioudakis, N. *et al.* Drivers of the summer-distribution of Northeast Atlantic mackerel (Scomber scombrus) in the Nordic Seas from 2011 to 2017; a Bayesian hierarchical modelling approach. *ICES J. Mar. Sci.* **76**, 530–548 (2018).

3. MacKenzie, B. R., Payne, M. R., Boje, J., Høyer, J. L. & Siegstad, H. A cascade of warming impacts brings bluefin tuna to Greenland waters. *Glob. Chang. Biol.* **20**, 2484–2491 (2014).

4. Muhling, B. A. *et al.* Projections of future habitat use by Atlantic bluefin tuna: Mechanistic vs. correlative distribution models. *ICES J. Mar. Sci.* **74**, 698–716 (2017).

5. Jansen, T. *et al.* Atlantic bluefin tuna (Thunnus thynnus) in Greenland – mixed-stock origin, diet, hydrographic conditions and repeated catches in this new fringe area. *Can. J. Fish. Aquat. Sci.* cjfas-2020-0156 (2020) doi:10.1139/cjfas-2020-0156.

6. Hátún, H., Payne, M. R. & Jacobsen, J. A. The North Atlantic subpolar gyre regulates the spawning distribution of blue whiting (Micromesistius poutassou). *Can. J. Fish. Aquat. Sci.* **66**, 759–770 (2009).

7. Pointin, F. & Payne, M. R. A Resolution to the Blue Whiting (Micromesistius poutassou) Population Paradox? *PLoS One* **9**, e106237 (2014).

8. Miesner, A. K. & Payne, M. R. Oceanographic variability shapes the spawning distribution of blue whiting (Micromesistius poutassou). *Fish. Oceanogr.* **27**, 623–638 (2018).

9. Bethke, I. *et al.* NorCPM1 and its contribution to CMIP6 DCPP. *Geosci. Model Dev. Discuss.* **2021**, 1–84 (2021).

10. Döscher, R. *et al.* The EC-Earth3 Earth System Model for the Climate Model Intercomparison Project 6. *Geosci. Model Dev. Discuss.* 1–90 (2021).

11. Tian, T. *et al.* Benefits of sea ice initialization for the interannual-to-decadal climate prediction skill in the Arctic in EC-Earth3. *Geosci. Model Dev.* **14**, 4283–4305 (2021).

12. Mauritsen, T. *et al.* Developments in the MPI-M Earth System Model version 1.2 (MPI-ESM1.2) and Its Response to Increasing CO2. *J. Adv. Model. Earth Syst.* **11**, 998–1038 (2019).

13. Müller, W. A. *et al.* A Higher-resolution Version of the Max Planck Institute Earth System Model (MPI-ESM1.2-HR). *J. Adv. Model. Earth Syst.* **10**, 1383–1413 (2018).

14. Williams, K. D. *et al.* The Met Office Global Coupled Model 3.0 and 3.1 (GC3.0 and GC3.1) Configurations. *J. Adv. Model. Earth Syst.* **10**, 357–380 (2018).

15. Yeager, S. G. *et al.* Predicting near-term changes in the earth system: A large ensemble of initialized decadal prediction simulations using the community earth system model. *Bull. Am. Meteorol. Soc.* **99**, 1867–1886 (2018).

16. Yeager, S. G. The abyssal origins of North Atlantic decadal predictability. *Clim. Dyn.* **55**, 2253–2271 (2020).
