# Supplementary material for: Skilful decadal-scale prediction of fish habitat and distribution shifts
Source: Nat Commun. 2022 May 12;13:2660. doi: 10.1038/s41467-022-30280-0 (PMC9098506; doi:10.1038/s41467-022-30280-0)
Supplement: Supplementary file 3 — Reporting Summary [file 41467_2022_30280_MOESM3_ESM.pdf]

Corresponding author(s): Mark R. Payne
Last updated by author(s): Apr 11, 2022

Reporting Summary

Nature Portfolio wishes to improve the reproducibility of the work that we publish. This form provides structure for consistency and transparency in reporting. For further information on Nature Portfolio policies, see our Editorial Policies and the Editorial Policy Checklist.

Statistics

For all statistical analyses, confirm that the following items are present in the figure legend, table legend, main text, or Methods section.

n/a	Confirmed	
☐	☒	The exact sample size (*n*) for each experimental group/condition, given as a discrete number and unit of measurement
☒	☐	A statement on whether measurements were taken from distinct samples or whether the same sample was measured repeatedly
☐	☒	The statistical test(s) used AND whether they are one- or two-sided<br>*Only common tests should be described solely by name; describe more complex techniques in the Methods section.*
☒	☐	A description of all covariates tested
☒	☐	A description of any assumptions or corrections, such as tests of normality and adjustment for multiple comparisons
☐	☒	A full description of the statistical parameters including central tendency (e.g. means) or other basic estimates (e.g. regression coefficient) AND variation (e.g. standard deviation) or associated estimates of uncertainty (e.g. confidence intervals)
☐	☒	For null hypothesis testing, the test statistic (e.g. F, t, r) with confidence intervals, effect sizes, degrees of freedom and P value noted<br>*Give P values as exact values whenever suitable.*
☒	☐	For Bayesian analysis, information on the choice of priors and Markov chain Monte Carlo settings
☒	☐	For hierarchical and complex designs, identification of the appropriate level for tests and full reporting of outcomes
☐	☒	Estimates of effect sizes (e.g. Cohen's *d*, Pearson's *r*), indicating how they were calculated

Our web collection on statistics for biologists contains articles on many of the points above.

Software and code

Policy information about availability of computer code

Data collection	No software was used.
Data analysis	Scripts used to generate these analyses are available online at https://github.com/markpayneatwork/PredictabilityEngine (doi:10.5281/zenodo.6451271)

For manuscripts utilizing custom algorithms or software that are central to the research but not yet described in published literature, software must be made available to editors and reviewers. We strongly encourage code deposition in a community repository (e.g. GitHub). See the Nature Portfolio guidelines for submitting code & software for further information.

Data

Policy information about availability of data

All manuscripts must include a data availability statement. This statement should provide the following information, where applicable:

- Accession codes, unique identifiers, or web links for publicly available datasets
- A description of any restrictions on data availability
- For clinical datasets or third party data, please ensure that the statement adheres to our policy

Climate predictions and projections analysed in this study are available from the CMIP data archives https://esgf-node.llnl.gov/projects/cmip6/. CESM-DPLE data are available from http://www.cesm.ucar.edu/projects/community-projects/DPLE/. HadISST data is available from https://www.metoffice.gov.uk/hadobs/hadisst/ and EN4 data from https://www.metoffice.gov.uk/hadobs/en4/. ICES stock assessment data is available from http://standardgraphs.ices.dk/.

Field-specific reporting

Please select the one below that is the best fit for your research. If you are not sure, read the appropriate sections before making your selection.

☒ Life sciences ☐ Behavioural & social sciences ☐ Ecological, evolutionary & environmental sciences

For a reference copy of the document with all sections, see nature.com/documents/nr-reporting-summary-flat.pdf

Life sciences study design

All studies must disclose on these points even when the disclosure is negative.

Sample size	Sample size was determined by the decadal prediction experiments that the authors could access . This is resulted in a total of 85 ensemble members. One ensemble member was chosen from each modelling centre in the CMIP6 historical experiment to produce an "uninitialised" ensemle, with the exclusions noted below.
Data exclusions	All ensemble members in the decadal prediction experiments were included. CMIP6 historical experiments that were not available on regular vertical levels were excluded from the "uninitialised" ensemble.
Replication	Replication of experiments was not attempted, due to the heavy computational nature of climate models.
Randomization	One level of grouping of decadal predictions was associated with the research institute performing the experiments. This was accounted for estimating and presenting the skill of each model system, as well as the ensemble mean.
Blinding	Blinding was not relevant to this study as all data as processed in a repeatible manner using scripts and therefore not subject to bias.

Reporting for specific materials, systems and methods

We require information from authors about some types of materials, experimental systems and methods used in many studies. Here, indicate whether each material, system or method listed is relevant to your study. If you are not sure if a list item applies to your research, read the appropriate section before selecting a response.

Materials & experimental systems

n/a	Involved in the study	
☒	☐	Antibodies
☒	☐	Eukaryotic cell lines
☒	☐	Palaeontology and archaeology
☒	☐	Animals and other organisms
☒	☐	Human research participants
☒	☐	Clinical data
☒	☐	Dual use research of concern

Methods

n/a	Involved in the study	
☒	☐	ChIP-seq
☒	☐	Flow cytometry
☒	☐	MRI-based neuroimaging
